# Diagnosis and Monitoring of Choroidal Osteoma through Multimodal Imaging

**DOI:** 10.1155/2014/393804

**Published:** 2014-09-08

**Authors:** Theodoros Empeslidis, Usman Imrani, Vasileios Konidaris, Fizza Mushtaq, Pandelis Fotiou, Periyasami Kumar, Somnath Banerjee, Konstantinos T. Tsaousis

**Affiliations:** Leicester Royal Infirmary, University Hospitals of Leicester, Ophthalmology Department, Medical Retina, Infirmary Square, Leicester, Leicestershire LE1 5WW, UK

## Abstract

A 16-year-old Caucasian female with a 6-month history of decreased visual acuity and metamorphopsia in the left eye is reported. The fundus of the left eye revealed a well defined lesion in the macula region. Diagnosis of choroidal osteoma was established using spectral domain optical coherence tomography (OCT), fundus fluorescein angiography (FFA), indocyanine green angiography (ICG), and B-scan ultrasonography. Subretinal fluid (SRF) and retinal pigment epithelium (RPE) detachment were noted in the absence of obvious classic choroidal neovascularisation (CNV). The patient was followed up for over 13 months without any treatment in the interim and the lesion was noted to have enlarged but visual acuity and SRF had remained stable. We report an interesting case where subretinal fluid was noted in the absence of evident choroidal neovascularisation and provide an example of the imaging modalities application in the era of “optical biopsy.”

## 1. Introduction

Choroidal osteoma was first described by Van Dyk at the Verhoeff society meeting in 1975 [1]. It is a benign ocular tumour of unknown aetiology characterised by the presence of cancellous bone within the choroid. It typically presents as a unilateral lesion in 75% of cases and commonly in healthy females in their 2nd or 3rd decades of life. The majority of cases discussed in the literature have been in Caucasians although there have been reports of patients of Afro-Caribbean and Oriental background [2–5].

There is no prevalence or incidence data in the literature and most papers consist of individual case reports, with the largest cohort consisting of 61 patients from ocular oncology service at Wills Eye Hospital, Thomas Jefferson University, Philadelphia, over a 26-year period [6]. Generally patients are asymptomatic at detection and the lesion is found incidentally. However, in those cases where symptoms are present the patient describes visual loss, metamorphopsia, and/or scotomas.

On fundoscopic investigation the choroidal osteoma is usually located in the peripapillary or juxtapapillary regions and may extend to the macula. Less commonly the mass may be found solely in the macula area [7, 8]. The colour of the lesion varies from yellow-white to orange-red with or without overlying clumps of pigment. This variation in colour is believed to relate to calcification of the osteoma, with orange-red appearance indicating a calcified tumour and white-yellow decalcification [9]. The shape is typically round or oval with well circumscribed borders.

Diagnostic approach involves multiple imaging modalities. Optical coherence tomography (OCT) may show areas of varying reflectivity depending on the calcification of the mass, with the decalcified portion more likely to be hyperreflective. It may also provide information regarding the status of the overlying retina and the presence of SRF. FFA typically shows that an early hyperfluorescent picture with a mottled appearance is seen [10]. This is followed by late and persisting diffuse hyperfluorescence. Presence of neovascularisation will lead to leakage of the fluorescein. ICG may show small feeder blood vessels on the anterior surface of the tumour during the early phases. These vessels may leak and are often not detected by FFA. The bony areas of the tumour show variable blockage of the choroidal vasculature [11]. Diagnosis may be confirmed by ultrasonography; a B-scan typically exhibits a slightly elevated choroidal mass with high reflectivity and acoustic shadowing giving a pseudooptic disc appearance.

As a consequence of the rarity, other ocular conditions must be considered when presented with the fundoscopic picture noted above. The list of differentials includes amelanotic choroidal melanoma/naevus, choroidal metastases, choroidal haemangioma, choroidal granuloma, and more.

## 2. Case Report

**Figure 1:**
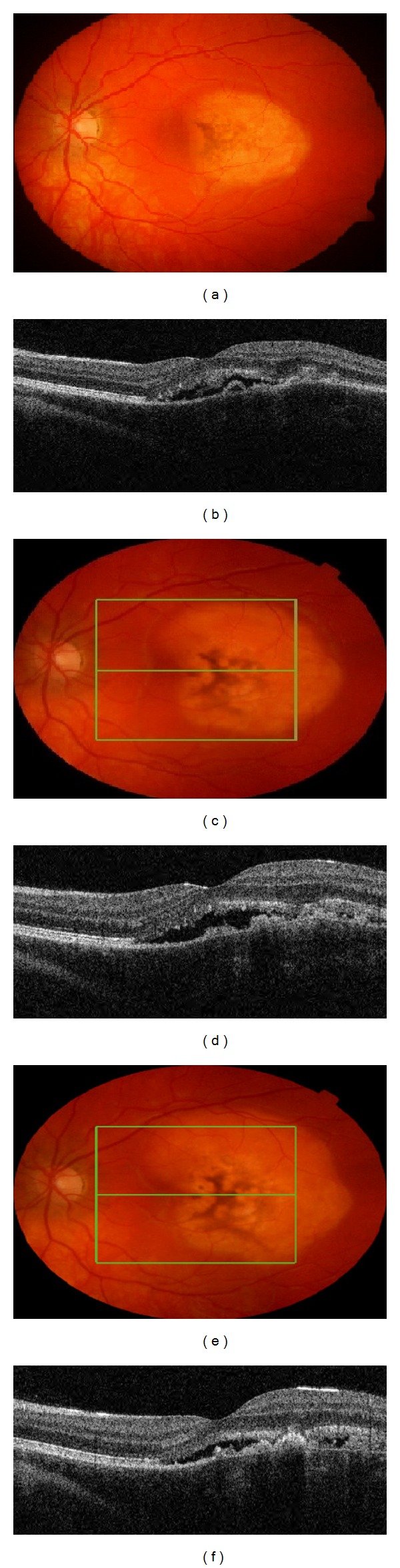
At presentation: (a) left eye fundus showing well circumscribed lesion at macula. (b) OCT of the left eye showing SRF and RPE detachment. First followup: (c) fundus, (d) OCT. Second followup: (e) fundus, (f) OCT.

A 16-year-old female was referred to clinic with a 6-month history of gradual decline in visual acuity and metamorphopsia. There was no relevant medical or previous ocular history. Visual acuity at presentation in the right eye was 6/6 and 6/18 in the left eye. Examination of the fundus revealed a well-defined orange lesion at the macula with overlying pigmentary changes (Figure 1(a)).

**Figure 2:**
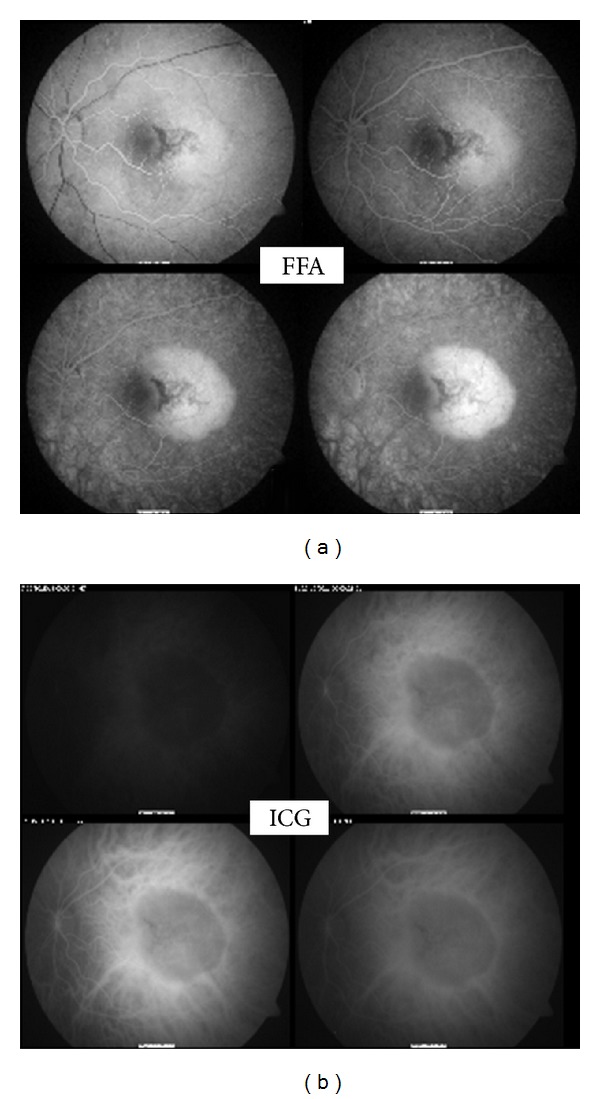
(a) FFA showing no evidence of CNV and late persisting hyperfluorescence. (b) ICG showing well defined choroidal mass.

**Figure 3:**
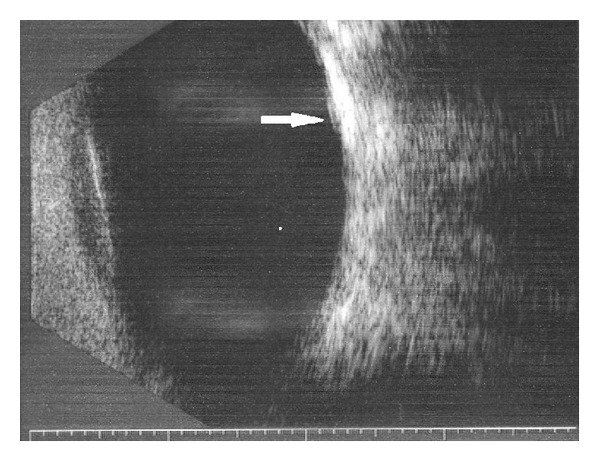
B-scan with white arrow indicating site of choroidal osteoma.

Spectral domain OCT (TopCon 3D 1000) revealed presence of SRF, a dome shaped RPE detachment with evidence of choroidal enlargement and a central retinal thickness of 297 microns (Figure 1(b)). FFA revealed RPE mottling, during the vein phase, a gradually increasing hyperfluorescence of a well demarcated lesion, and late persisting hyperfluorescence with no evidence of pinpoint leakage or late phase leakage. This increasing hyperfluorescence appears to be occurring within the entire tumor with possibly some pooling under the subretinal space. (Figure 2(a)). ICG reveals also a well-defined mass and no evidence of hot spots, staining, or leakage of the inner choroid (Figure 2(b)). Finally, B-scan provided the information to confirm the clinical diagnosis. An elevated choroidal mass of high reflectivity and acoustic shadowing was noted (pseudooptic disc) (Figure 3).

The patient was referred to a specialist ocular oncologist and following assessment it was decided that active intervention was not currently appropriate and regular followup was the preferred management plan.

The patient was seen in clinic for followup 7 months later. Patient reported that visual acuity remained stable but metamorphopsia was still present. On clinical examination visual acuity remained stable at 6/18. Fundoscopic examination revealed that the osteoma had grown superiorly and temporally (Figure 1(c)). OCT showed that SRF was still present, with an increase in central retinal thickness to 340 microns (Figure 1(d)). A hyperreflective spot posterior to the SRF was noted and corresponded to atrophy. It was decided that management would remain conservative with followup in a further 6 months.

The patient was again seen for followup a further 6 months later. The patient reported visual acuity and metamorphopsia remained stable. Visual acuity was again recorded at 6/18. The osteoma continued to grow in a superotemporal direction (Figure 1(e)). OCT revealed that SRF was still present but central retinal thickness decreased to 251 microns (Figure 1(f)). Although anti-VEGF therapy could be a reasonable option at this point, the conservative approach of close followup was selected, mostly because of the lack of progression in symptoms and the stability of central retinal thickness.

## 3. Discussion

In the case discussed above, it is postulated that subretinal fluid has accumulated due to the concurrent RPE detachment and RPE dysfunction. The RPE has many physiological roles; one of these is to transport fluid produced by the metabolically active retina into the choriocapillaris [12]. Thus derangement of the RPE function at the site of detachment may have led to the accumulation of the subretinal fluid. This accumulation could also be attributed to an occult choroidal neovascular membrane or leakage from the growing tumors vessels.

Morbidity in choroidal osteoma is a consequence of SRF accumulation, haemorrhage from choroidal neovascularisation, or degeneration of the overlying RPE or sensory retina. Prognosis is relatively poor in the affected eye. In a long term follow up study by Shields et al. in 61 patients; tumour growth was noted in 51% of cases, decalcification in 50% and visual acuity of 20/200 or less was found in 56% of cases at 10 years [6]. However, tumour growth appears to halt when decalcification occurs. Another long term followup by Aylward et al., of 36 patients, found tumour growth in 41% and loss of visual acuity to 20/200 or less in 58% at 10 years [5]. However, due to the large majority of cases being unilateral the patients generally maintain good vision in the unaffected eye.

Treatment options for foveal choroidal osteoma are limited (PDT is a reasonable choice in the case of extrafoveal lesions). Observation is the indicated management where there are no symptoms, with fundus examination at regular intervals monitoring for signs of CNV. In the past photocoagulation has been used to treat choroidal osteoma related CNV [13]. Whilst this effectively sealed new vessels, there was limited improvement in visual acuity. Verteporfin ocular photodynamic therapy (PDT) has also been utilised in the treatment of choroidal osteoma related CNV. Parodi et al. reported a case where a patient refused treatment of extrafoveal CNV via photocoagulation and PDT was specifically requested [14]. Visual acuity stabilised, symptoms of metamorphopsia settled, and CNV resolved. Shields et al. also reported a case of extrafoveal CNV successfully treated with PDT [15]. However, the author inserted a proviso at the end of the case report that treatment of subfoveal CNV with PDT may result in worse visual acuity due to decalcification and associated RPE loss. More recently antivascular endothelial growth factor (anti-VEGF) drugs have been used off license to treat CNV secondary to choroidal osteoma with good effect. Ahmadieh and Vafi (2007) reported a case where VA improved from 20/200 to 20/20 using bevacizumab [16]. Another case was reported by Morris et al., where VA improved from 20/80 to 20/20 using combination of PDT and Ranibizumab [17]. A one-year followup of CNV secondary to choroidal osteoma was conducted by Wu et al. [18]. They reported improvement in VA from 20/800 to 20/30 following treatment with ranibizumab with no further decline in VA after treatment. Reports of management using PDT and anti-VEGF are promising and a recent study demonstrated that antivascular endothelial growth factor treatment alone or with PDT had a favoured outcome in the anatomy of the area and modest improvement in visual acuity [19]. Further large scale studies are required to determine the efficacy of PDT and anti-VEGF drugs in the treatment of choroidal osteoma related CNV.

To conclude, choroidal osteoma is a rare benign ossifying tumour of the choroid. The clinical picture may appear as a malignant ocular tumour. Understanding the disease and how to appropriately investigate those who present it are important to avoid incorrect diagnosis. We present a case with SRF in the absence of CNV which is documented through multiple imaging modalities. We have monitored the patient for more than a year and found that there has been no significant progression in SRF, development of CNV, or decrease in visual acuity despite the continued growth of the osteoma. Therefore, the need for active management may not be required in cases similar to these.
